# Increased susceptibility to oral *Trichuris muris* infection in the specific absence of CXCR5^+^ CD11c^+^ cells

**DOI:** 10.1111/pim.12566

**Published:** 2018-07-13

**Authors:** Barry M. Bradford, David S. Donaldson, Ruth Forman, Kathryn J. Else, Neil A. Mabbott

**Affiliations:** ^1^ The Roslin Institute & Royal (Dick) School of Veterinary Sciences University of Edinburgh Edinburgh UK; ^2^ Faculty of Biology, Medicine and Health University of Manchester Manchester UK

**Keywords:** CXCR5, dendritic cell, helminth, mucosal immunity, Th2, *Trichuris muris*

## Abstract

*Trichuris muris* is a natural mouse helminth pathogen which establishes infection specifically in the caecum and proximal colon. The rapid expulsion of *T. muris* in resistant mouse strains is associated with the induction of a protective T helper cell type 2 (Th2)‐polarized immune response. Susceptible mouse strains, in contrast, mount an inappropriate Th1 response to *T. muris* infection. Expression of the chemokine CXCL13 by stromal follicular dendritic cells attracts CXCR5‐expressing cells towards the B‐cell follicles. Previous studies using a complex in vivo depletion model have suggested that CXCR5‐expressing conventional dendritic cells (cDC) help regulate the induction of Th2‐polarized responses. Here, transgenic mice with CXCR5 deficiency specifically restricted to CD11c^+^ cells were used to determine whether the specific absence CXCR5 on CD11c^+^ cells such as cDC would influence susceptibility to oral *T. muris* infection by affecting the Th1/Th2 balance. We show that in contrast to control mice, those which lacked CXCR5 expression on CD11c^+^ cells failed to clear *T. muris* infection and developed cytokine and antibody responses that suggested a disturbed Th1/Th2 balance with enhanced IFN‐γ expression. These data suggest an important role of CXCR5‐expressing CD11c^+^ cells such as cDC in immunity to oral *T. muris* infection.

## 1 INTRODUCTION

Mononuclear phagocytes (MNP) arise from precursors in the bone marrow and comprise a heterogeneous population of monocytes, conventional dendritic cells (cDC) and tissue macrophages. The intestinal mucosa is populated by distinct MNP populations including MNP expressing the fractalkine receptor CX3CR1 and subsets of cDC marked by differential expression of the integrins CD11b and CD103.^1^ The cDC are specialized antigen‐presenting cells, and antigen presentation by cDC to uninfected cognate CD4^+^ T cells may induce T helper cell type 1 (Th1), Th2 or Th17 responses dependent on their subset.^2^, ^3^, ^4^, ^5^, ^6^ The development of the intestinal cDC1 subclass of CD103^+^CD11b^−^ cDC is dependent on the transcription factors IRF8, BATF3 and ID2.^7^, ^8^, ^9^, ^10^ In contrast, the lack of IRF4 or Notch2 results in a loss of cDC2 subclass CD103^+^CD11b^+^ cDC and reduced numbers of CD103^−^CD11b^+^ cDC in the intestine‐draining mesenteric lymph nodes (MLN).^3^, ^4^, ^5^, ^6^

*Trichuris muris* is a natural nematode parasite of mice whose larvae hatch in the caecum and proximal colon and invade the epithelium. Resistance to high‐level infection with *T. muris* varies considerably between different conventional mouse strains. In resistant mouse strains, the rapid expulsion of *T. muris* before the adult worms reach fecundity is associated with the induction a protective Th2‐polarized immune response characterized by the production of the cytokines interleukin (IL)‐4, IL‐5, IL‐9 and IL‐13.^11^, ^12^, ^13^, ^14^ In contrast, susceptible mouse strains mount an inappropriate Th1‐polarized response to *T. muris* infection that is associated with high levels of IFN‐γ and IL‐12, and results in susceptibility and persistent infection.^15^, ^16^

While the development of Th1 immunity is well understood and regulated by cDC‐derived production of the cytokine interleukin (IL)‐12, the factors that regulate the development of Th2 immunity are less clear. Expression of the chemokine CXCL13 by stromal follicular dendritic cells (FDC) and follicular stromal cells mediates the attraction of CXCR5‐expressing cells, including cDC, towards and into the B‐cell follicles.^17^, ^18^, ^19^, ^20^ A requirement for CXCR5‐expressing cDC has been suggested for the efficient development of Th2 responses to the intestinal parasite *Heligmosomoides polygyrus*.^21^ This evidence, however, was derived from the use of a complex irradiation chimeric mouse model. Briefly, C57BL/6 wild‐type mice were first lethally γ‐irradiated and reconstituted with an 80:20 mixture of bone marrow from CD11c‐DTR mice (in which CD11c^+^ cells can be transiently ablated by diphtheria toxin treatment^22^) and *Cxcr5* ^−/−^ mice. After reconstitution, purified uninfected CD4^+^T cells were then transferred into these chimeric mice before they were infected with *H. polygyrus*. Data from the use of these “DC‐*Cxcr5* ^−/−^” chimeric mice suggested that CXCR5‐expressing cDC helps regulate the induction of Th2‐polarized responses.^21^ However, all the MNP populations in the intestine are transiently depleted in CD11c‐DTR mice after diphtheria toxin treatment.^23^ This may have influenced disease susceptibility as intestinal macrophages also contribute to immunity to *H. polygyrus* infection.^24^ It is also plausible that the use of lethal irradiation may have adversely affected gut integrity and the microarchitecture of the secondary lymphoid organs.

Whether CXCR5‐expressing cDC are important for the induction of protective immunity to other helminth pathogens such as *T. muris* was not known. Therefore, in the current study, a novel compound transgenic mouse model was used in which CXCR5 deficiency was specifically restricted to CD11c^+^ cells, including cDC.^25^ These mice were used to test the hypothesis that CXCR5‐expressing CD11c^+^ cells such as cDC are required for the induction of protective immune responses to *T. muris* infection.

## 2 MATERIALS AND METHODS

### 2.1 Mice

The following mouse strains were used in this study where indicated: CD11c‐Cre^26^ (strain Tg(Itgax‐cre)1‐1Reiz) and CXCR5^F/F^ (strain Cxcr5^tm1.Namt^), which have *loxP* sites flanking exon 2 of the *Cxcr5* gene.^25^ All mice were bred and maintained on C57BL/6J mice background, maintained under SPF conditions and used at 8‐12 weeks of age. All studies and regulatory licences were approved by the University of Edinburgh's Ethics Committee and carried out under the authority of a UK Home Office Project Licence. The genotypes of all mice used in this study were confirmed by the analysis of genomic or cDNA extracted from ear punch biopsies. DNA samples were analysed for the presence of CD11c‐Cre using the following primers: ACTTGGCAGCTGTCTCCAAG and GCGAACATCTTCAGGTTCTG; and CXCR5^F^ and recombined CXCR5^F^ (Cxcr5^de‐flox^) using the following primers: AGGAGGCCATTTCCTCAGTT; GGCTTAGGGATTGCAGTCAG; and TTCCTTAGAGCCTGGAAAAGG.

### 2.2 Trichuris muris infection

Mice (n* *=* *8/group) were infected by oral gavage with approximately 200 embryonated E isolate *T. muris* eggs suspended in H_2_O. Mice were killed at various times after infection and the worm burden in the large intestine assessed as previously described.^27^

### 2.3 Quantitative real‐time reverse transcriptase PCR (qRT‐PCR)

Mesenteric lymph nodes (MLN) were snap‐frozen in liquid nitrogen. Samples were homogenized using a FastPrep 24 and lysing matrix D (MP Biomedicals, Illkirch, France) and total RNA extracted using RNABee (AmsBio, Abingdon, UK). The total RNA concentration was measured by absorbance at 260 nm on a NanoDrop ND‐1000 spectrophotometer (Labtech International, East Sussex, UK). Samples were treated with RNase‐free DNase (Promega, Southampton, UK) to remove any contaminating genomic DNA. Total RNA (1.0 μg) was then reverse‐transcribed using SuperScript® III First‐Strand Synthesis System for RT‐PCR (Life Technologies, Waltham, MA, USA) in a final volume of 50 μL according to the manufacturer's instructions. qRT‐PCR was performed using FastStart Universal SYBR Green Master (Rox) (Sigma‐Aldrich, Poole, Dorset, UK) and the primers listed in Table 1 on an MX3005P qPCR machine (Agilent Technologies LDA UK Ltd, Stockport, Cheshire, UK) with MxPro software (Agilent Genomics). Expression levels were determined relative to *Rpl19* expression using the ΔΔCt method. Gene expression data were then normalized so that the mean expression level of each gene of interest in uninfected CXCR5^F/F^ control mice was 1.0.

**Table 1:** qRT‐PCR primer pairs used

Target gene	3′ primer	5′ primer
*Ifng*	TGAGCTCATTGAATGCTTGG	ACAGCAAGGCGAAAAAGGAT
*Il1b*	CTGAACTCAACTGTGAAATGCCA	AAAGGTTTGGAAGCAGCCCT
*Il4*	CGAGCTCACTCTCTGTGGTG	TGAACGAGGTCACAGGAGAA
*Il5*	CCCACGGACAGTTTGATTCT	GCAATGAGACGATGAGGCTT
*Il6*	ACCAGAGGAAATTTTCAATAGGC	TGATGCACTTGCAGAAAACA
*Il9*	CATCAGTGTCTCTCCGTCCCAAC	GATTTCTGTGTGGCATTGGTCAG
*Il12a*	GCTTCTCCCACAGGAGGTTT	CTAGACAAGGGCATGCTGGT
*Il12b*	GAGACACCAGCAAAACGAT	GATTCAGACTCCAGGGGACA
*Il13*	CACACTCCATACCATGCTGC	TGTGTCTCTCCCTCTGACCC
*Il17a*	TGGTTCATTATTCGGGCAAT	GCCACATTAAAGACAAAGAAGGT
*Rpl19*	GAAGGTCAAAGGGAATGTGTTCA	CCTTGTCTGCCTTCAGCTTGT
*Tnfa*	AGGGTCTGGGCCATAGAACT	CCACCACGCTCTTCTGTCTAC

### 2.4 Parasite‐specific immunoglobulin (Ig) ELISA

Serum *T. muris* antigen‐specific IgG1 and IgG2c levels were determined by ELISA as previously described.^28^ Nunc™ MaxiSorp™ uncoated 96‐well ELISA plates (Thermo Fisher Scientific) were first coated overnight at 4°C with 5 μg/mL (50 μL/well) *T. muris* excretory/secretory (E/S) antigen^27^ or polyclonal goat anti‐mouse Ig antibody (BD Pharmingen), each diluted in 0.05M carbonate/bicarbonate buffer (pH9.6; 1.59 g/L Na2CO3, 2.93 g/L NaHCO3). Plates were subsequently incubated with serum samples diluted through eight serial doubling dilutions from 1/20 to 1/2560. Purified mouse IgG1, κ isotype [clone MG1‐45] (Biolegend, San Diego, CA, USA), or purified mouse IgG2a, κ isotype [clone MG2a‐53] (Biolegend), was used as controls. Parasite‐specific immunoglobulin was detected using either biotin rat anti‐mouse IgG1 [clone A85‐1] (BD Pharmingen) or biotin rat anti‐mouse IgG2a/c [clone R19‐15] (BD Pharmingen). Bound Ig was detected using streptavidin/POD conjugate (Sigma‐Aldrich) and TMB SureBlue substrate (KPL Inc. Lubbox, TX, US). Reactions stopped with 75 μL 1N HCl, and absorbance at 450 nm and 595 nm plates read on a Wallac plate reader.

### 2.5 Histopathology and immunohistochemistry (IHC)

Intestines, MLN and spleens were snap‐frozen at the temperature of liquid nitrogen. Serial frozen sections (10 μm) were cut on a cryostat and immunostained with the following antibodies: Alexa Fluor® 488 anti‐mouse CD4 antibody [clone RM4‐5] (Life Technologies), Alexa Fluor® 488 anti‐mouse CD8a antibody [clone 53‐6.7] (Biolegend), Alexa Fluor® 594 anti‐mouse/human CD11b antibody [clone M1/70] (Biolegend), purified hamster anti‐mouse CD11c [clone N418] (AbD Serotec) detected with Biotin‐SP (long spacer) AffiniPure goat anti‐Armenian hamster IgG (H+L) (Jackson ImmunoResearch) and Alexa Fluor® 594 tyramide (Life Technologies), Alexa Fluor® 647 anti‐mouse CD11c antibody [clone N418] (Biolegend), purified rat anti‐mouse CD35 [clone 8C12] (BD Pharmingen) detected with Alexa Fluor® 488 goat anti‐rat IgG (H+L) cross‐adsorbed secondary antibody (Life Technologies), Alexa Fluor® 594 anti‐mouse CD45 antibody [clone 30‐F11] (Biolegend), Alexa Fluor® 488 anti‐mouse/human CD45R/B220 antibody [clone RA3‐6B2] (Biolegend) and Alexa Fluor® 488 anti‐mouse CD68 antibody [clone FA‐11] (Biolegend). Sections were imaged using a Zeiss LSM710 confocal microscope (Zeiss, Welwyn Garden City, UK).

Intestines were also immersion‐fixed in 10% neutral‐buffered formalin and embedded into paraffin wax. Paraffin‐embedded sections (6 μm) were dewaxed in xylene and alcohol, and mucin detected using a periodic acid/Schiff (PAS) stain kit (Mucin Stain) (Abcam), according to the manufacturer's instructions. Sections were imaged using a Nikon Eclipse Ni‐U bright‐field microscope (Nikon UK Limited).

### 2.6 Image analysis

For morphometric analysis, images were analysed using ImageJ software (http://rsb.info.nih.gov/ij/) as described on coded sections.^29^ Crypt and cell counting were performed manually using the FiJi cell counter plug‐in. In each instance, data were typically obtained from 14 to 37 crypts/mouse, from the intestines of 4 to 8 mice/group. Details of the sample sizes for each parameter analysed are provided in the figure legends.

### 2.7 Statistical analysis

Unless indicated otherwise, data are presented as mean ± SEM and significant differences between groups were sought using Student's *t*‐test and ANOVA with Tukey's post hoc grouping test for those with a standard distribution. In instances where there was evidence of non‐normality (identified by the Kolmogorov‐Smirnov test), a Mann‐Whitney *U* test or Kruskal‐Wallis test was used. Values of *P *<* *0.05 were accepted as significant. Data analyses were performed using GraphPad Prism 6.04 software (GraphPad Software, La Jolla, CA, USA).

## 3 RESULTS

### 3.1 Altered positioning of CD11c^+^ cells in the secondary lymphoid organs of CXCR5^ΔDC^ mice

**Figure 1:**
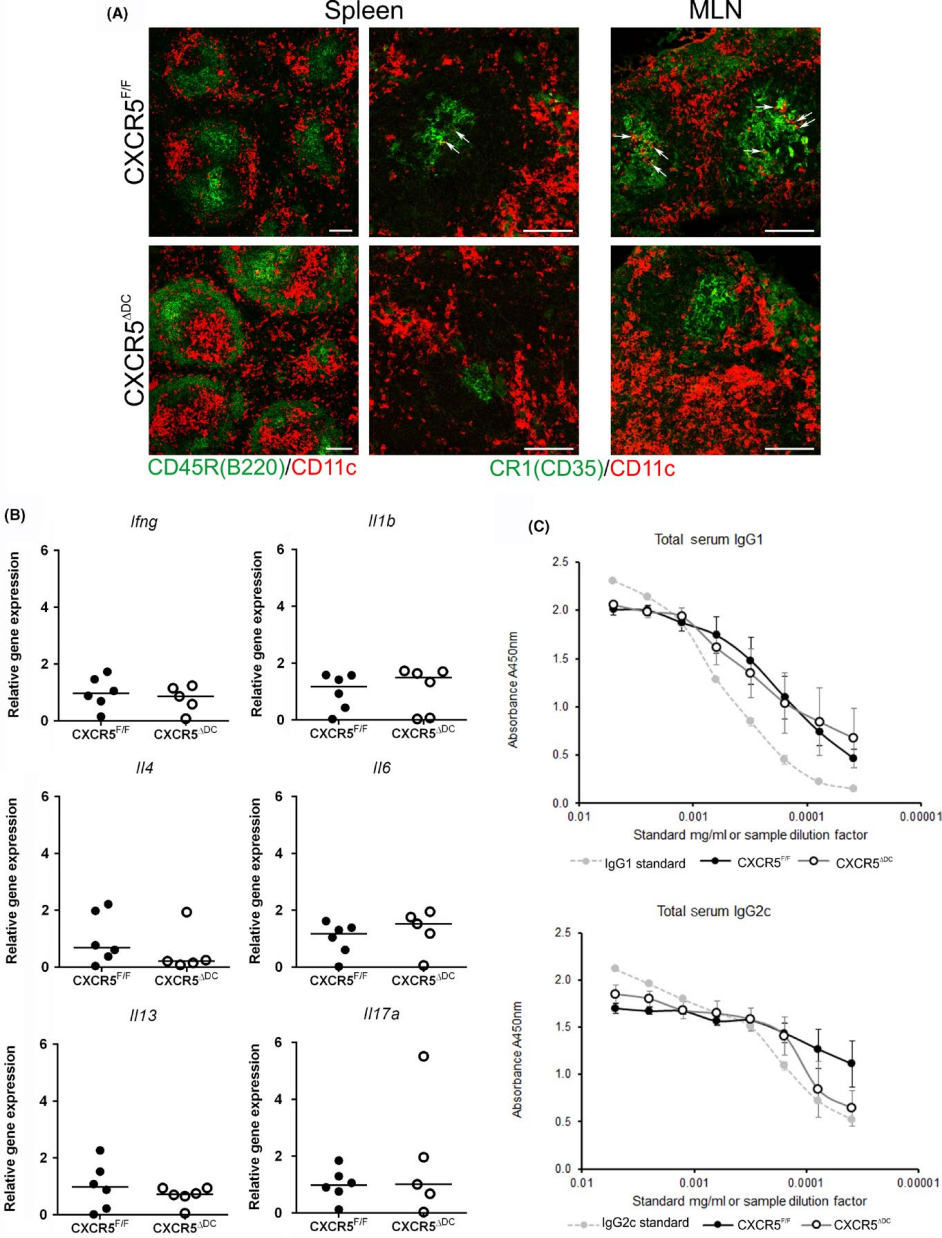
Altered positioning of CD11c^+^ cells in the secondary lymphoid organs of CXCR5^Δ^ ^DC^ mice. A, Immunohistochemical (IHC) analysis of the distribution of B cells (CD45R/B220^+^  cells; green, left‐hand column), stromal FDC (CR1/CD35^+^ cells; green) and CD11c^+^ cells (red) in the spleen and MLN of uninfected CXCR5^F/F^ control mice (upper panels) and CXCR5^Δ^ ^DC^ mice (lower panels). In the MLN and spleens of CXCR5^F/F^ mice, CD11c^+^ cells were occasionally detected within the FDC‐containing B‐cell follicles (arrows). Few, if any, CD11c^+^ cells were detected in the FDC‐containing B‐cell follicles of CXCR5^Δ^ ^DC^ mice. Scale bar, 100 μm. B, qRT‐PCR analysis of cytokine‐encoding genes in mRNA from the MLN of uninfected CXCR5^F/F^ control mice (closed circles) and CXCR5^Δ^ ^DC^ mice (open circles). Horizontal bars, median. C, Similar levels of total IgG1 and IgG2c were detected in the serum of uninfected CXCR5^F/F^ control mice (closed circles) and CXCR5^Δ^ ^DC^ mice (open circles). Data were derived from 6 mice/group

Throughout this study, a novel compound transgenic mouse model was used in which CXCR5 deficiency was specifically restricted to CD11c^+^ MNP.^25^ The expression of Cre recombinase under the control of the *Itgax* locus (encoding CD11c) in CD11c‐Cre mice has been used in a variety of studies to conditionally control gene expression in cDC.^3^, ^25^, ^26^, ^30^, ^31^ These mice were crossed to CXCR5^F/F^ mice to generate CXCR5^ΔDC^ mice.^25^ We have previously shown that in these mice, the Cre recombinase‐mediated recombination of *Cxcr5* is restricted to CD11c^+^ cDC and that the migration of their cDC towards CXCL13 is specifically impeded.^25^ In the MLN and spleens of CXCR5^F/F^ control mice, CD11c^+^ cells were occasionally detected within the FDC‐containing B‐cell follicles (Figure 1A, arrows). However, in tissues from CXCR5^ΔDC^ mice, few, if any, CD11c^+^ cells were detected in the FDC‐containing B‐cell follicles (Figure 1A), consistent with the impaired ability of the cDC in these mice to migrate towards CXCL13.^25^

### 3.2 Cytokine mRNA levels are not altered in the MLN of CXCR5^ΔDC^ mice in the steady state

Assessment of the steady‐state mRNA expression levels of cytokine‐encoding genes in the MLN of CXCR5^ΔDC^ mice revealed similar levels of *Il4* and *Il13* expression when compared to CXCR5^F/F^ mice, suggesting no differences in the expression of steady‐state Th2 cytokine levels (Figure 1B). The expression of genes encoding the Th1 cytokine INF‐γ, the proinflammatory cytokines IL‐1β and IL‐6, and the Th17 cytokine IL‐17 was also similar in MLN from CXCR5^F/F^ mice CXCR5^ΔDC^ mice (Figure 1B). Therefore, no significant differences in the expression of steady‐state Th1, Th2 and Th17 cytokines were observed in the MLN of uninfected CXCR5^ΔDC^ mice when compared to uninfected CXCR5^F/F^ mice.

### 3.3 Steady‐state serum IgG1 and IgG2c Ig levels are not altered in CXCR5^ΔDC^ mice

Next, the relative concentrations of total IgG1 and IgG2c Ig isotype levels were compared in the serum of uninfected CXCR5^F/F^ mice and CXCR5^ΔDC^ mice. Similar levels of total IgG1 (*P *=* *0.590) and IgG2c (*P *=* *0.946) were detected in mice from each group, indicating no constitutive difference in the ability to produce either Ig isotype in the steady state (Figure 1C).

### 3.4 Enhanced susceptibility to *T. muris* infection in CXCR5^ΔDC^ mice

**Figure 2:**
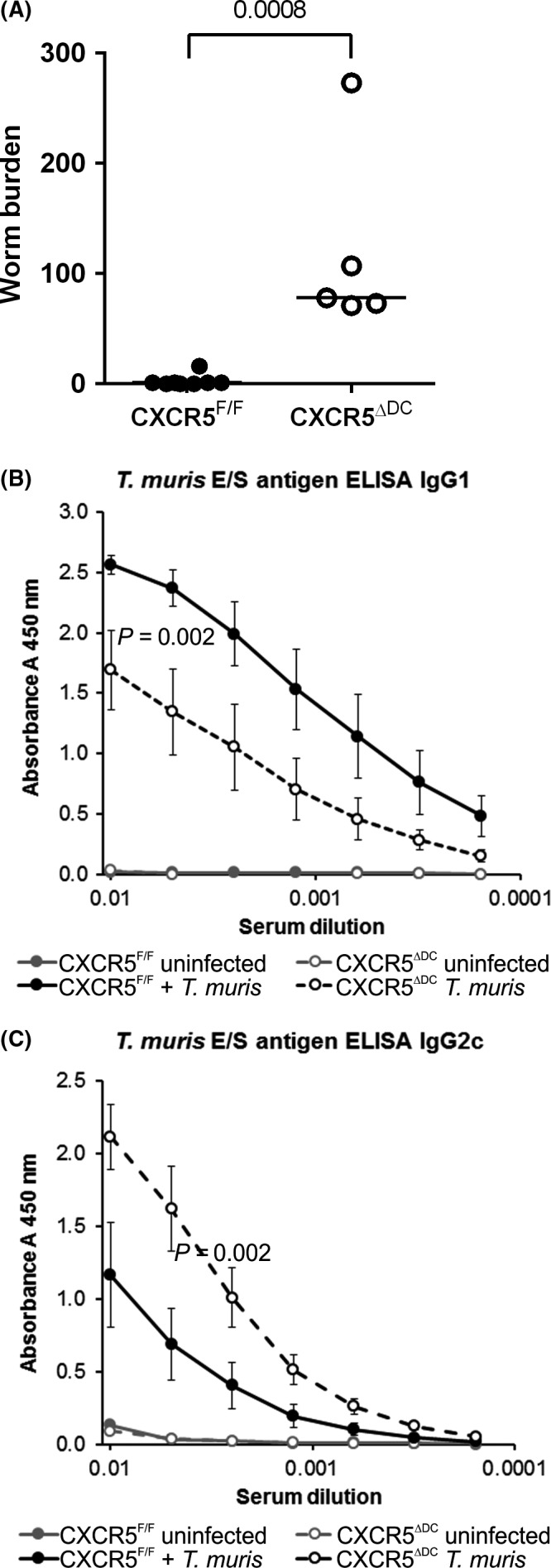
Susceptibility to *Trichuris muris* infection is enhanced in the specific absence of CXCR5‐expressing cDC in CXCR5^Δ^ ^DC^ mice. CXCR5^F/F^ mice (n* *=* *8, closed circles) and CXCR5^Δ^ ^DC^ mice (n* *=* *5, open circles) were orally infected with approximately 200 embryonated *T. muris* eggs and worm burdens in the large intestine compared at 30 dpi. Susceptibility to *T. muris* infection was significantly increased in CXCR5^Δ^ ^DC^ mice when compared to CXCR5^F/F^ control mice (*P *<* *0.008). (B&C) Serum *T. muris* E/S antigen‐specific IgG1 (B) and IgG2c (C) levels were determined by ELISA. When compared to *T. muris*‐infected CXCR5^F/F^ mice, the sera of *T. muris*‐infected CXCR5^Δ^ ^DC^ mice contained significantly lower levels of parasite‐specific IgG1 (B; *P *<* *0.002) and significantly higher levels of IgG2c (C; *P *<* *0.002)

Groups of CXCR5^F/F^ mice and CXCR5^ΔDC^ mice were next orally infected with approximately 200 embryonated *T. muris* eggs. Post‐mortem analysis of the large intestines of *T. muris*‐infected sentinel animals at 14 days post‐infection (dpi) confirmed the successful establishment of infection in mice of each genotype (data not shown). Resistant background mouse strains such as C57BL/6 used here typically expel *T. muris*, whereas susceptible strains retain adult worms in the intestine by 30 dpi. As anticipated, at 30 dpi the worm burdens in the large intestines of CXCR5^F/F^ control mice were predominantly low (Figure 2A; median 1, range 0‐16, n* *=* *8), consistent with the previous data. In comparison, susceptibility to *T. muris* infection was significantly increased in CXCR5^ΔDC^ mice (*P *<* *0.0008, Mann‐Whitney *U* test). The large intestines of all the CXCR5^ΔDC^ mice were chronically infected at 30 dpi with large numbers of adult worms present in the caecum and proximal colon (Figure 2A; median 78, range 71‐274, n* *=* *5).

### 3.5 *T. muris*‐specific antibody responses are altered in CXCR5^ΔDC^ mice

Infection and expulsion of *T. muris* infection in resistant mouse strains are associated with the production of a strong parasite‐specific antibody response, the nature of which is indicative of the Th1/Th2 balance and the degree of susceptibility to the infection.^32^ In the sera of *T. muris*‐infected CXCR5^F/F^ control mice, high levels of parasite‐specific IgG1 and low levels of IgG2c isotype antibodies were detected, consistent with the induction of a Th2‐polarized antibody response observed in relatively resistant C57BL/6 mice. In contrast, the sera of *T. muris*‐infected CXCR5^ΔDC^ mice contained significantly lower levels of parasite‐specific IgG1 (Figure 2B; *P *<* *0.002) and significantly higher levels of IgG2c (Figure 2C; *P *<* *0.002). This parasite‐specific Ig profile in the sera of *T. muris*‐infected CXCR5^ΔDC^ mice was similar to that observed in other susceptible mouse strains and indicative of induction of an impaired Th2‐polarized immune response and enhanced Th1 environment.^15^, ^32^ Parasite‐specific IgG1 and IgG2c were undetectable in the sera of uninfected mice from both genotypes as expected (Figure 2B,C, respectively).

### 3.6 Altered expression of Th1/Th2 cytokines in the MLN of *T. muris*‐infected CXCR5^ΔDC^ mice

**Figure 3:**
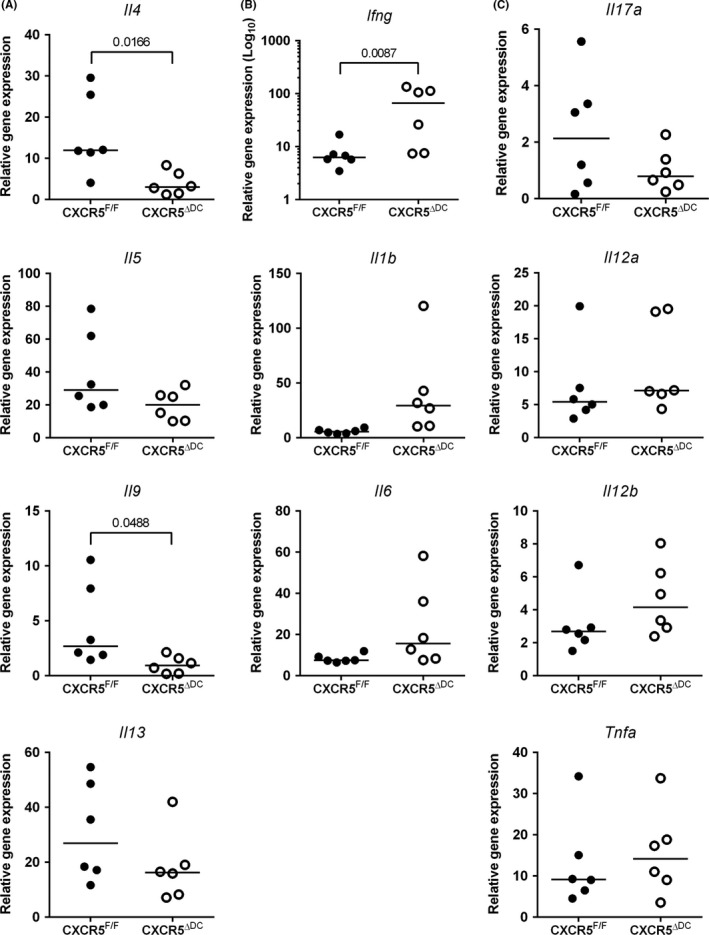
The expression of Th1/Th2 cytokines in the MLN following *Trichuris muris* infection is altered in the specific absence of CXCR5‐expressing cDC. CXCR5^F/F^ mice (n* *=* *6) and CXCR5^Δ^ ^DC^ mice (n* *=* *6) were orally infected with approximately 200 embryonated *T. muris* eggs, and at 30 dpi, the expression of cytokine‐encoding genes in the MLN was compared by qRT‐PCR analysis. A, Comparison of the expression of mRNA encoding the Th2 cytokines IL‐4, IL‐5, IL‐9 and IL‐13. B, Comparison of the expression of mRNA encoding the Th1 cytokine IFN‐γ (*Ifng*) and proinflammatory cytokines IL‐1β (*Il1b*) and IL‐6. C, The expression levels of *Il17a* (encoding IL‐17) and D, *Il21a*,* Il12b* and *Tnfa* (encoding the IL‐12p35 and IL‐12p40 subunits, and TNF‐α, respectively) were similar in MLN from *T. muris*‐infected CXCR5^F/F^ mice and CXCR5^Δ^ ^DC^ mice. Gene expression data show the relative expression level in infected mice compared to uninfected CXCR5^F/F^ control mice. Data were normalized so that the mean level in uninfected CXCR5^F/F^ control mice was 1.0. Horizontal bars, median. Data were derived from MLN from 6 mice/group. CXCR5^F/F^ mice (closed circles) and CXCR5^Δ^ ^DC^ mice (open circles)

The increased susceptibility of CXCR5^ΔDC^ mice to oral *T. muris* infection and their increased production of parasite‐specific serum IgG2c suggested an altered Th1/Th2 balance. We therefore compared the expression of cytokine‐encoding genes in mRNA from the MLN of *T. muris*‐infected CXCR5^F/F^ mice and CXCR5^ΔDC^ mice. The resistance of C57BL/6 mice to *T. muris* infection is associated with the induction of a Th2‐polarized parasite‐specific immune response. As anticipated, the expression of mRNA encoding the Th2 cytokines IL‐4, IL‐5, IL‐9 and IL‐13 was upregulated in the MLN of *T. muris*‐infected CXCR5^F/F^ control mice when compared to uninfected mice (Figure 3A). In contrast, the expression of *Il4* and *Il9* mRNA in the MLN of chronically infected CXCR5^ΔDC^ mice was significantly less increased when compared to uninfected control mice (Figure 3A).

In the MLN of *T. muris*‐infected CXCR5^F/F^ control mice, only modest increases in mRNA encoding the Th1 cytokine IFN‐γ and the proinflammatory cytokines IL‐1β and IL‐6 were observed (Figure 3B). In contrast, the expression of *Ifng* was substantially and significantly elevated in the MLN of infected CXCR5^ΔDC^ mice (*P *<* *0.0087, Mann‐Whitney *U* test; Figure 3B). These data indicated that in the absence of CXCR5‐expressing cDC, the Th1/Th2 cytokine balance in the MLN was disturbed with significantly reduced expression of IL‐4 and IL‐9 and significantly elevated expression of IFN‐γ. The expression levels of *Il12a*,* Il12b* and *Tnfa* (encoding the IL‐12p35 and IL‐12p40 subunits, and TNF‐α, respectively) and *Il17a* were similar in MLN from *T. muris*‐infected CXCR5^F/F^ mice and CXCR5^ΔDC^ mice (Figure 3C).

### 3.7 Goblet cell hyperplasia is unaltered in the proximal colon of *T. muris*‐infected CXCR5^ΔDC^ mice

**Figure 4:**
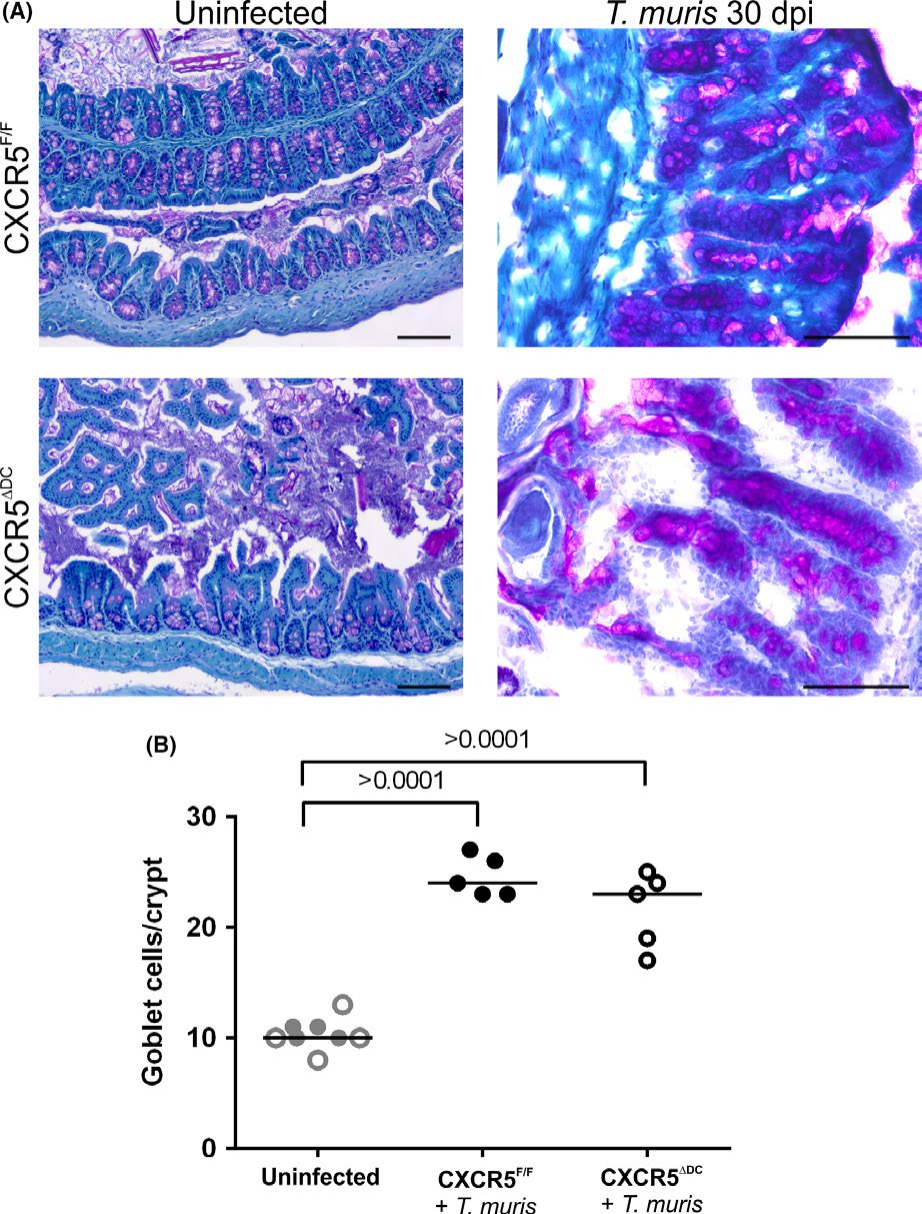
Goblet cell hyperplasia is unaltered in the proximal colons of *Trichuris muris*‐infected CXCR5^Δ^ ^DC^ mice. A, Histological analysis of mucous‐secreting goblet cells (pink) in PAS‐stained sections from the colons of uninfected and *T. muris*‐infected CXCR5^F/F^ mice and CXCR5^Δ^ ^DC^ mice. Sections were counterstained with haematoxylin (blue). Scale bar, 100 μm. B, Following *T. muris* infection, a significant increase in goblet cell density was observed in the large intestines of CXCR5^F/F^ mice (black closed circles) when compared to uninfected controls (grey closed circles). A similar significant increase in goblet cell density was also observed in *T. muris*‐infected CXCR5^Δ^ ^DC^ mice (infected CXCR5^Δ^ ^DC^ mice, black open circles; uninfected CXCR5^Δ^ ^DC^ mice, grey open circles), which was not significantly different from that observed in *T. muris*‐infected CXCR5^F/F^ mice. Data are derived from 14 to 37 crypts/mouse, from the intestines of 5‐8 mice/group. Horizontal bars, median. Data were analysed by ANOVA with Tukey's post hoc grouping

Goblet cells in the epithelium of the large intestine produce a range of effector molecules which are important for innate defence against helminth infections.^33^ Expansion of goblet cells and their production of mucins and other effector molecules contributes to the expulsion of *T. muris* infection.^34^ In the steady state, the densities of goblet cells within the crypts were similar in the large intestines of uninfected CXCR5^F/F^ mice and CXCR5^ΔDC^ mice (Figure 4). Following *T. muris* infection, a statistically significant increase in goblet cell density was observed in the large intestines of CXCR5^F/F^ mice (Figure 4B; *P *<* *0.001, ANOVA with Tukey's post hoc grouping). A similar increase in goblet cell density following *T. muris* infection was also observed in CXCR5^ΔDC^ mice, revealing that the ability to induce goblet cell hyperplasia was unaffected.

### 3.8 Influence of cDC‐specific CXCR5 deficiency on the abundance of leucocytes in the large intestinal lamina propria

**Figure 5:**
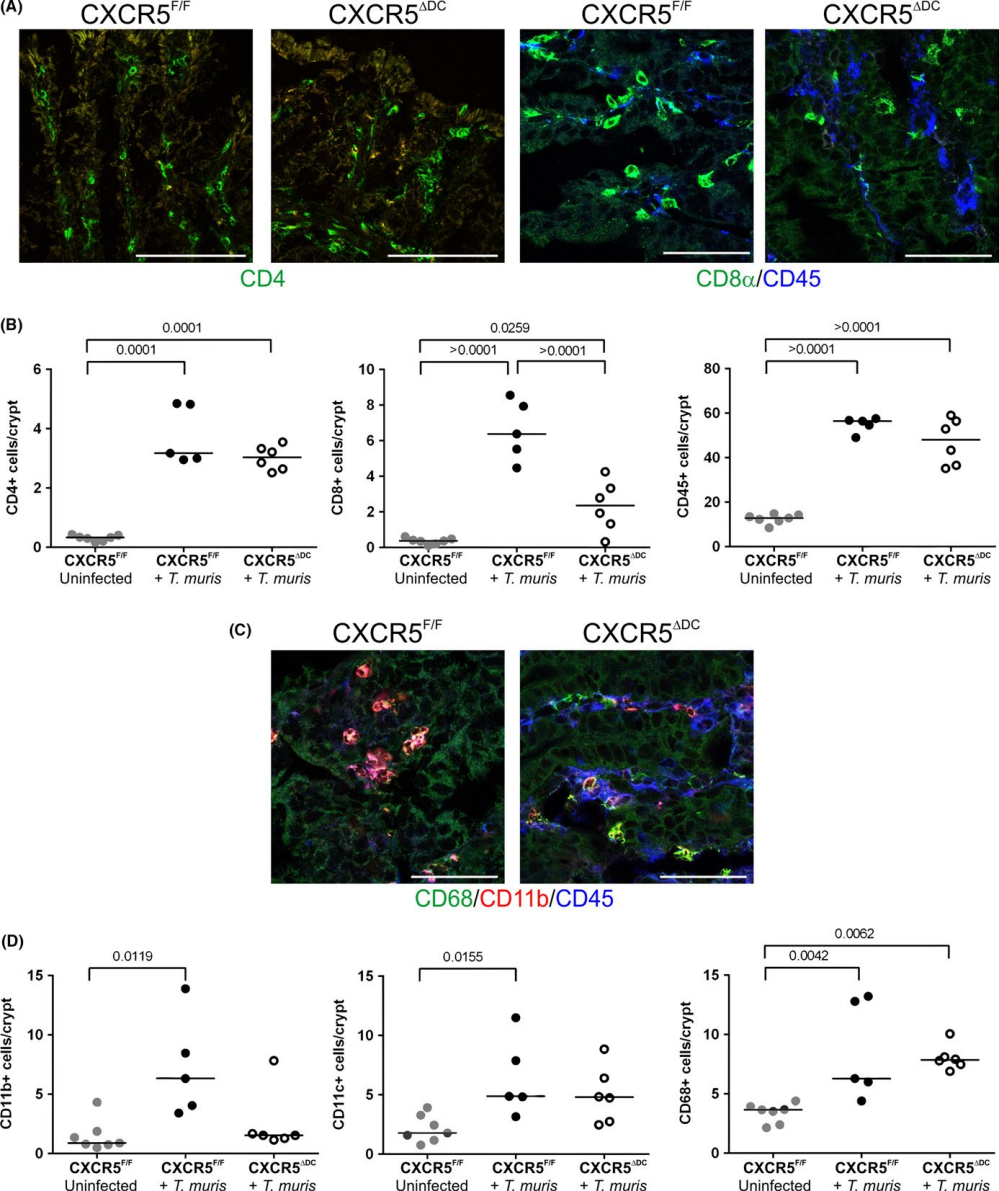
Influence of cDC‐specific CXCR5 deficiency on the abundance of leucocytes in the large intestinal lamina propria. A, IHC analysis of CD45^+^ cells (blue) and CD4^+^ lymphocytes (green) and CD8α^+^ lymphocytes in the lamina propria of the large intestines of *Trichuris muris*‐infected CXCR5^F/F^ mice and CXCR5^Δ^ ^DC^ mice. B, Dot plots show the numbers of CD45^+^ cells, CD4^+^ lymphocytes and CD8α^+^ lymphocytes/crypt. Horizontal bars, median. *T. muris*‐infected CXCR5^F/F^ mice (closed circles) and *T. muris*‐infected CXCR5^Δ^ ^DC^ mice (open circles). C, IHC analysis of the abundance of CD11b^+^ (red), CD11c^+^ (blue) and CD68^+^ mononuclear phagocytes in the lamina propria of the large intestines of *T. muris*‐infected CXCR5^F/F^ mice and CXCR5^Δ^ ^DC^ mice. D, Dot plots show the numbers of CD11b^+^, CD11c^+^ and CD68^+^ mononuclear phagocytes/crypt. Horizontal bars, median. *T. muris*‐infected CXCR5^F/F^ mice (closed circles) and *T. muris*‐infected CXCR5^Δ^ ^DC^ mice (open circles). Data are derived from 12 to 29 crypts/mouse, from the intestines of 5‐7 mice/group. Scale bars, 100 μm. All data were analysed by ANOVA with Tukey's post hoc grouping, with the exception of the abundance of CD11b^+^ cells which was analysed by Kruskal‐Wallis test

Leucocytes accumulate in the lamina propria of the large intestine during *T. muris* infection, and the characteristics of the response can differ between resistant and susceptible mouse strains.^35^ We therefore compared the density of T cells and MNP within the lamina propria in the large intestines of *T. muris*‐infected CXCR5^F/F^ mice and CXCR5^ΔDC^ mice (Figure 5). Although similar levels of CD4^+^ lymphocytes were detected in the lamina propria of infected CXCR5^F/F^ mice and CXCR5^ΔDC^ mice, the number of CD8α^+^ lymphocytes in the lamina propria of CXCR5^ΔDC^ mice was significantly reduced (Figure 5A,B). Comparison of densities of CD11b^+^, CD11c^+^ and CD68^+^ cells suggested that similar densities of CD68^+^ MNP were present in the large intestines of *T. muris*‐infected CXCR5^F/F^ mice and CXCR5^ΔDC^ mice (Figure 5C,D). However, the density of CD11b^+^ and CD11c^+^ MNP in *T. muris*‐infected CXCR5^ΔDC^ mice was similar to uninfected CXCR5^F/F^ mice (Figure 5C,D).

## 4 DISCUSSION

The induction of a parasite‐specific Th2‐polarized immune response associated with the production of IL‐4, IL‐5, IL‐9 and IL‐13 is important for the expulsion of *T. muris* infection in resistant mouse strains.^11^, ^12^, ^13^, ^14^ Susceptible mouse strains, in contrast, elicit an inappropriate Th1‐polarized immune response associated with high levels of IFN‐γ.^15^, ^16^ The precise mechanisms that regulate the induction of a protective immune response to *T. muris* infection are uncertain. Our data suggest an important role for CXCR5‐expressing CDllc^+^ cells such as cDC in the induction of a protective immune response to *T. muris* infection. In the specific absence of CXCR5^+^ CDllc^+^ cells, the mice were unable to expel *T. muris* infection, exhibited a disturbed Th1/Th2 cytokine balance in their MLN and had reduced serum levels of parasite‐specific IgG1. The cytokine IL‐4 is an Ig subclass‐switching factor for IgG1, whereas IFN‐γ promotes Ig subclass switching to IgG2a/c.^36^, ^37^ The significantly reduced serum levels of parasite‐specific IgG1 and enhanced levels of parasite‐specific IgG2c in infected CXCR5^ΔDC^ mice were consistent with the reduced expression of IL‐4 and enhanced IFN‐γ expression in their MLN, supporting the suggestion of a disturbed Th1/Th2 cytokine balance in the MLN of CXCR5^ΔDC^ mice. Although CD4^+^ Th1 and Th2 cells are credible sources of the IFN‐γ and IL‐4 (respectively) detected in the current study, their precise identity was not addressed. Further experiments are required to exclude the contribution of CD8^+^ T cells as potential additional sources as IFN‐γ and innate lymphoid cells, basophils and eosinophils as sources of IL‐4.

Murine cDC have often been discriminated based on their expression of high levels of the integrin CD11c. However, the expression of this integrin is not only restricted to cDC. Other MNP populations including certain macrophage populations and activated monocytes can also express CD11c.^23^, ^38^ In the intestinal lamina propria, there is an almost complete overlap between the expression of the macrophage‐specific F4/80 Ag and CD11c, and each completely overlaps with macrophage colony‐stimulating factor 1 receptor (CSF1R) expression.^23^ Furthermore, the treatment of CD11c‐DTR‐tg mice with diphtheria toxin transiently ablates all the MNP in the intestine. Thus, although our data support a role of CXCR5‐epxressing CD11c^+^ cells such as cDC in protective immunity against oral *T. muris* infection, a role of other MNP such as macrophages in this process cannot be entirely excluded. Subsets of other immune cell populations such as germinal centre B cells may also express CD11c under certain circumstances^39^; thus, more refined transgenic mouse models are required to study the specific role of cDC. Expression of the zinc finger transcription factor ZBTB46 is restricted to pre‐DC and their progeny.^40^, ^41^ The generation of *Zbtb46*‐driven Cre transgenic mice^42^, ^43^ provides an excellent opportunity to further define the role of CXCR5‐expressing cDC in immunity to *T. muris* infection.

The cDC in the intestine can be subdivided based on their expression of the integrins CD11b and CD103. Mice with cDC‐specific deficiency in the IRF8 transcription factor lack intestinal CD103^+^CD11b^−^ cDC in their MLN and the intestinal lamina propria and fail to induce effective Th1‐polarized immune responses.^7^ CD103^+^ cDC are recruited to the colon in response to *T. muris* infection.^44^ However, these Th1‐polarizing cDC are dispensable for immunity to *T. muris* infection,^45^, ^46^ consistent with the requirement for the induction of a protective parasite‐specific Th2‐polarized immune response. CD103^−^CD11b^+^ cDC, in contrast, are important for the induction of Th2 responses in the large intestine.^47^, ^48^ Mice deficient in IRF4 or Notch2 have reduced numbers of CD103^−^CD11b^+^ cDC and fail to mount Th2 responses to helminth infections including *T. muris*.^47^ Interestingly, our retrospective analysis of microarray data of mRNA derived from mesenteric lymph‐draining intestinal CD103^−^CD11b^+^ cDC and CD103^+^CD11b^+^ cDC^47^ shows that *Cxcr5* is selectively expressed by the CD103^−^CD11b^+^ cDC subset and enhanced after treatment with *Schistosoma mansoni* egg antigens which are potent stimulators of Th2 responses (Figure S1). Further studies are required to determine whether the effects observed in the current study are due to the expression of CXCR5 in a specific cDC populations such as the cDC1,^7^, ^8^, ^9^, ^10^ cDC2^3^, ^4^, ^5^, ^6^ or other MNP^49^, ^50^ subsets.

The secretion of mucous by goblet cells plays a key role in the expulsion of intestinal helminths.^33^ Expression of the Th2 cytokines IL‐4 and IL‐13 is considered important for the induction of goblet cell hyperplasia during helminth infections.^51^ In the current study, goblet cell hyperplasia was unaffected in the large intestines of *T. muris*‐infected CXCR5^ΔDC^ mice, despite the impaired expression of IL‐4 in their MLN. Thus, although the Th1/Th2 balance was disturbed in *T. muris*‐infected CXCR5^ΔDC^ mice, these data suggest that the remaining Th2 response was sufficient to induce the goblet cell hyperplasia. Data suggest that IL‐22 also plays a central role in promoting goblet cell abundance and function during helminth infection.^34^ Expression of IL‐22 alone may be sufficient to enhance mucin production by the intestinal epithelium,^34^ suggesting a potential mechanism through which this response may be maintained. Although similar levels of goblet cell hyperplasia were observed in the intestines of infected CXCR5^ΔDC^ mice and CXCR5^F/F^ mice, the CXCR5^ΔDC^ mice were unable to clear the worm infection. This indicates that goblet cell hyperplasia on its own is insufficient to expel the worms from the intestine. Treatment of B‐cell‐deficient μMT mice with purified *T. muris*‐specific IgG1 has been reported to restore resistance to infection.^52^ Thus, it is plausible that the reduced serum levels of parasite‐specific IgG1 may contribute to the increased susceptibility of CXCR5^ΔDC^ mice to *T. muris* infection.

Studies have suggested that cDC can capture and retain unprocessed antigens, transfer them to naive B cells and provide signals to the B cells that can modulate and influence the subclass of the immunoglobulin response.^53^, ^54^ Furthermore, interactions between B cells and IL‐12‐expressing cDC have been shown to enhance the ability to induce Th2 differentiation.^55^ Consistent with these observations, susceptibility to *T. muris* infection is increased in B‐cell‐deficient μMT mice and coincides with reduced expression of Th2 cytokines and enhanced expression of IFN‐γ in their MLN.^52^ CXCR5 mediates the migration of cells towards the chemokine CXCL13 produced by stromal FDC and follicular stromal cells in the B‐cell follicle.^17^, ^18^, ^19^, ^20^ Our data imply that the migration of cDC towards and/or within the B‐cell follicle is important for them to effectively induce a protective Th2‐polarized immune response against infection with *T. muris*. The requirement for effective Th2 priming by CXCR5‐expressing cDC is not restricted to *T. muris* infection, as a similar requirement during infection with *H. polygyrus* and the intracellular parasite *Leishmania major* has been described.^21^ A thorough understanding of the role of CXCR5‐expressing cDC in the induction of Th2 responses may identify novel therapeutic targets to enhance immunity to helminths or modulate the pathology caused by certain allergic inflammatory diseases.

## CONFLICT OF INTEREST

The authors declare no competing conflict of interests.

## AUTHOR CONTRIBUTIONS

BB, DD, KE and NM conceived and designed the study; BB and DD performed the study; BB, DD, RF, KE and NM analysed and interpreted the data; BB, DD, RF, KE and NM wrote the manuscript.
