# Supplementary material for: Increased susceptibility to oral Trichuris muris infection in the specific absence of CXCR5+ CD11c+ cells
Source: Parasite Immunol. 2018 Jul 13;40(8):e12566. doi: 10.1111/pim.12566 (PMC6099414; doi:10.1111/pim.12566)
Supplement: Supplementary file 1 [file PIM-40-na-s001.docx]

**Increased susceptibility to oral *Trichuris muris* infection in the specific absence of CXCR5-expressing dendritic cells**

Barry M. Bradford, David S. Donaldson, Ruth A. Forman, Kathryn J. Else & Neil A. Mabbott

**
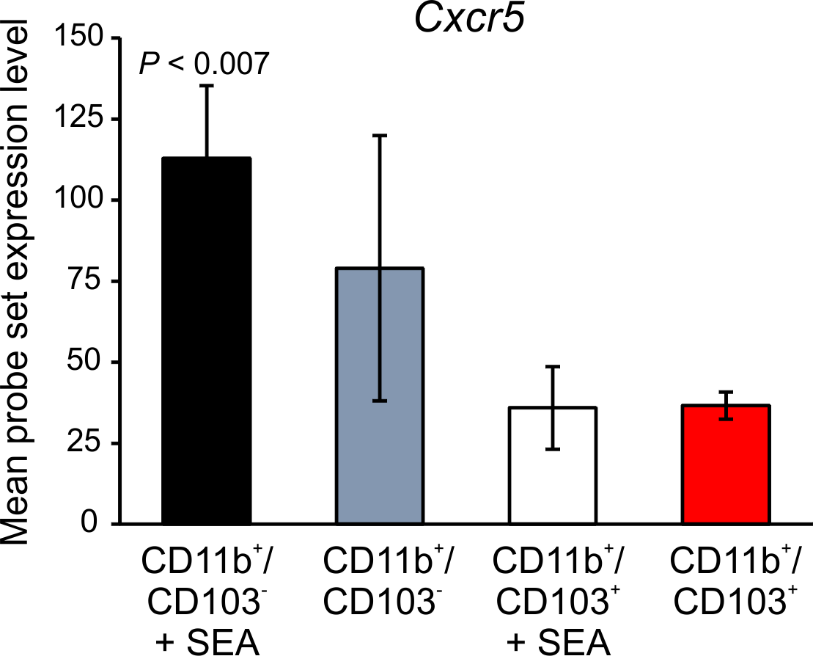
**

**Supplementary Figure 1.** Comparison of *Cxcr5* expression in mRNA from intestinal CD11b^+^/CD103^-^ and CD11b^+^/CD103^+^ lymph draining cDC. Analysis of *Cxcr5* mRNA expression levels in microarray data derived sorted mesenteric lymph-draining intestinal CD11b^+^/CD103^-^ and CD11b^+^/CD103^+^ cDC populations ([1]; GSE91381). Cells (30,000) were purified by FACS and incubated *in vitro* with/without soluble *Schistosoma mansoni* egg antigen (SEA) for 18 h before analysis [1]. Lymph-draining intestinal CD11b^+^CD103^-^ (single-positive) cDC selectively expressed *Cxcr5*, and was significantly increased following exposure to SEA. Data represent mean of 3 data sets/group ± SD.

1. Mayer JU, Demiri M, Agace WW, MacDonald AS, Svensson-Frej M, Milling SW. Different populations opf CD11b^+^ dendritic cells drive Th2 responses in the small intestine and colon. *Nat Commun* 2017; **8:** 15820.
